# Supplementary material for: Development of a Novel Biomarker for the Progression of Idiopathic Pulmonary Fibrosis
Source: Int J Mol Sci. 2024 Jan 2;25(1):599. doi: 10.3390/ijms25010599 (PMC10779374; doi:10.3390/ijms25010599)
Supplement: Supplementary file 1 [file ijms-25-00599-s001.zip › ijms-2760073-supplementary.pdf]

Supplementary files

Development of a Novel Biomarker for the Progression of Idiopathic Pulmonary Fibrosis

Hye Ju Yeo [1,2,3,†], Mihyang Ha [4,5,†], Dong Hoon Shin [3,6], Hye Rin Lee [3], Yun Hak Kim [7,8,*] and Woo Hyun Cho [1,2,3,*]

1 Department of Internal Medicine, School of Medicine, Pusan National University, Yangsan 50612, Republic of Korea; dugpwn@naver.com
2 Division of Pulmonary, Allergy, and Critical Care Medicine, Department of Internal Medicine, Pusan National University Yangsan Hospital, Yangsan 50612, Republic of Korea
3 Research Institute for Convergence of Biomedical Science and Technology, Pusan National University Yangsan Hospital, Yangsan 50612, Republic of Korea; donghshin@chol.com (D.H.S.); hrlee01070@gmail.com (H.R.L.)
4 Interdisciplinary Program of Genomic Data Science, Pusan National University, Busan 46241, Republic of Korea; mh2059389@naver.com
5 Department of Nuclear Medicine, Pusan National University Medical Research Institute, Pusan National University Hospital, Busan 49241, Republic of Korea
6 Department of Pathology, School of Medicine, Pusan National University, Yangsan 50612, Republic of Korea
7 Department of Anatomy, School of Medicine, Pusan National University, Yangsan 50612, Republic of Korea
8 Department of Biomedical Informatics, School of Medicine, Pusan National University, Yangsan 50612, Republic of Korea
* Correspondence: yunhak10510@pusan.ac.kr (Y.H.K.); chowh@pusan.ac.kr (W.H.C.); Tel.: +82-51-510-8091 (Y.H.K.); +82-55-360-2120 (W.H.C.); Fax: +82-51-510-8049 (Y.H.K.); +82-55-360-2157 (W.H.C.)
† These authors contributed equally to this work.

Table S1. List of 16 common DEGs.

Gene (Official symbol)	Full name
**Up-regulated genes**	
ALDH16A1	Aldehyde Dehydrogenase 16 Family Member A1
CD276	CD276 Molecule
CLEC11A	C-Type Lectin Domain Containing 11A
COL7A1	Collagen Type VII Alpha 1 Chain
CTSB	Cathepsin B
FDXR	Ferredoxin Reductase
GLI2	GLI Family Zinc Finger 2
HOMER3	Homer Scaffold Protein 3
PIK3R2	Phosphoinositide-3-Kinase Regulatory Subunit 2
PRAF2	PRA1 Domain Family Member 2
SNED1	Sushi, Nidogen And EGF Like Domains 1
TCIRG1	T Cell Immune Regulator 1, ATPase H+ Transporting V0 Subunit A3
TYMS	Thymidylate Synthetase
**Down-regulated genes**	
**Gene (Official symbol)**	**Full name**
ADAMTS8	ADAM Metallopeptidase With Thrombospondin Type 1 Motif 8
GRIA1	Glutamate Ionotropic Receptor AMPA Type Subunit 1
SGMS1	Sphingomyelin Synthase 1

DEGs: differentially expressed genes.

Table S2. ANOVA or Kruskal-Wallis rank sum test result.

Kruskal-Wallis rank sum test				ANOVA test							
GSE10667				GSE24206				PNU			
**Gene**	**Group**	**Median±IQR**	**H(p)**	**Gene**	**Group**	**Mean±SD**	**F(p)**	**Gene**	**Group**	**Mean±SD**	**F(p)**
ALDH16A1	Control^{a}	2378.3±1658.4	9.003*	***ALDH16A1***	Control^{a}	7.1±0.2	6.159**	***ALDH16A1***	Control^{a}	3.2±1.6	6.499*
	E^{b}	3426.7±1456.4			E^{ab}	7.5±0.2			E^{ab}	5.6±2.3	
	A^{b}	3583.2±1048.1			A^{b}	7.7±0.4			A^{b}	10.5±3.5	
**CD276**	Control^{a}	129.5±208.2	6.585*	**CD276**	Control^{a}	6.2±0.2	6.597**	**CD276**	Control	9.4±4.5	5.326*
	E^{b}	268.5±167.0			E^{ab}	6.5±0.3			E	10.0±9.2	
	A^{ab}	287.2±87.7			A^{b}	6.8±0.3			A	31.1±12.3	
CLEC11A	Control^{a}	817.7±712.8	15.910***	***CLEC11A***	Control^{a}	6.4±0.2	6.188**	***CLEC11A***	Control	2.8±1.3	3.029*
	E^{b}	1773.1±1163.3			E^{b}	7.0±0.4			E	6.2±2.0	
	A^{b}	2400.7±1796.6			A^{b}	7.0±0.4			A	14.9±10.5	
COL7A1	Control^{a}	364.1±245.1	20.348***	***COL7A1***	Control^{a}	6.2±0.2	3.985*	***COL7A1***	Control^{a}	2.0±1.0	7.096*
	E^{b}	1126.9±972.2			E^{ab}	6.9±0.7			E^{ab}	7.2±4.9	
	A^{b}	1386.4±1265.2			A^{b}	7.1±0.6			A^{b}	18.2±7.9	
CTSB	Control^{a}	8913.5±4609.9	17.214***	***CTSB***	Control^{a}	11.1±0.6	6.442**	***CTSB***	Control^{a}	55.4±24.0	7.666*
	E^{b}	17727.3±12890.7			E^{b}	11.7±0.3			E^{ab}	74.7±55.2	
	A^{b}	20901.4±13388.9			A^{b}	11.8±0.4			A^{b}	225.7±81.2	
FDXR	Control^{a}	4769.8±3028.1	15.686***	***FDXR***	Control^{a}	5.7±0.4	17.247***	***FDXR***	Control^{a}	1.3±1.1	8.322*
	E^{b}	8680.5±6613.2			E^{b}	6.6±0.4			E^{a}	2.0±1.3	
	A^{b}	9891.5±3499.9			A^{b}	6.9±0.4			A^{b}	5.7±1.7	
GLI2	Control^{a}	155.4±151.9	16.743***	***GLI2***	Control^{a}	5.7±0.2	4.782*	***GLI2***	Control^{a}	1.4±0.5	7.015*
	E^{b}	344.8±213.8			E^{ab}	5.9±0.3			E^{ab}	1.7±1.7	
	A^{b}	377.8±99.0			A^{b}	6.2±0.4			A^{b}	5.3±1.8	
HOMER3	Control^{a}	8407.8±5084.9	22.051***	***HOMER3***	Control^{a}	6.3±0.1	3.785*	***HOMER3***	Control^{a}	5.3±1.9	27.23**
	E^{b}	17238.5±7418.5			E^{ab}	6.5±0.3			E^{a}	9.1±3.2	
	A^{b}	18219.0±7417.9			A^{b}	6.6±0.2			A^{b}	18.2±1.0	
PIK3R2	Control^{a}	43.7±46.4	8.556*	***PIK3R2***	Control^{a}	6.8±0.2	4.990*	***PIK3R2***	Control^{a}	3.1±1.9	12.62**
	E^{b}	93.7±35.2			E^{ab}	7.0±0.2			E^{a}	3.1±1.2	
	A^{b}	109.0±75.2			A^{b}	7.1±0.2			A^{b}	12.0±3.8	
PRAF2	Control^{a}	832.6±530.2	13.448**	***PRAF2***	Control^{a}	7.9±0.3	3.745*	***PRAF2***	Control	5.5±2.6	6.494*
	E^{b}	1350.3±462.4			E^{ab}	8.1±0.4			E	5.6±3.5	
	A^{b}	1400.0±554.7			A^{b}	8.3±0.3			A	17.2±6.6	
SNED1	Control^{a}	405.5±267.3	16.301***	***SNED1***	Control	6.6±0.4	3.850*	***SNED1***	Control^{a}	11.4±7.7	7.668*
	E^{b}	950.6±609.5			E	6.7±0.4			E^{a}	12.1±4.6	
	A^{b}	1260.2±599.1			A	7.1±0.3			A^{b}	26.5±2.4	
TCIRG1	Control	11364.5±7582.7	6.355*	***TCIRG1***	Control^{a}	8.7±0.3	5.154*	***TCIRG1***	Control^{a}	23.4±8.8	5.588*
	E	15735.5±6370.4			E^{ab}	9.3±0.4			E^{ab}	31.6±15.0	
	A	16267.7±3431.2			A^{b}	9.4±0.5			A^{b}	53.5±9.4	
TYMS	Control^{a}	340±227.7	19.334***	***TYMS***	Control^{a}	6.0±0.2	14.794***	***TYMS***	Control^{a}	1.4±0.8	24.136**
	E^{b}	988.1±566.6			E^{b}	6.7±0.3			E^{a}	4.3±1.9	
	A^{b}	1280.1±486.0			A^{b}	6.8±0.3			A^{b}	9.5±1.5	
ADAMTS8	Control^{b}	1287.2±1276.2	10.326**	**ADAMTS8**	Control^{b}	7.8±0.7	5.192*	**ADAMTS8**	Control	17.0±6.7	6.602*
	E^{ab}	919.8±632.8			E^{a}	7.0±0.5			E	5.2±4.3	
	A^{a}	298.6±377.9			A^{a}	6.9±0.5			A	5.1±0.4	
GRIA1	Control^{b}	79.9±98.5	15.237***	**GRIA1**	Control^{b}	6.2±0.8	8.198**	**GRIA1**	Control^{b}	6.0±2.4	14.918**
	E^{a}	22.4±25.6			E^{a}	5.3±0.5			E^{a}	0.9±0.2	
	A^{a}	14.4±49.3			A^{a}	5.0±0.4			A^{a}	0.5±0.3	
SGMS1	Control^{b}	1596.2±769	6.187*	**SGMS1**	Control^{b}	8.9±0.4	4.196*	**SGMS1**	Control^{b}	27.8±5.3	9.397*
	E^{ab}	1289±615.4			E^{ab}	8.8±0.4			E^{ab}	18.9±3.0	
	A^{a}	1041.4±486.9			A^{a}	8.4±0.3			A^{a}	15.4±1.6	

P values were obtained from ANOVA test followed by Scheffe post-hoc test or Kruskal-Wallis rank sum test followed by Bonferroni's multiple comparison test; ANOVA test on GSE24206 and PNU datasets, Kruskal-Wallis rank sum test on GSE10667 dataset. "*", "**" and "***" indicate $p<0.05$, $p<0.01$ and $p<0.001$, respectively. $^{a-b}$: Same letters in the same column indicate no statistically significant differences. While, different letters in the same column indicate significant statistical differences. E: Early IPF; A: advanced IPF; IPF: Idiopathic pulmonary fibrosis.

Table S3. Patient characteristics of the test cohort.

	Control (n=29)	NP (n=24)	P (n=25)	p
Age (years)	48.9 ± 8.4	66.6 ± 9.0	64.6 ± 5.7	<0.001***
Male	15 (78.9)	22 (91.7)	17 (68.0)	0.007**
BMI (kg/m^2)	23.4±2.8	23.5±4.2	22.0±3.6	0.241
Smoking history				
Never smoking		5 (20.8)	8 (32.0)	0.376
Former smoker		19 (79.2)	17 (68.0)	
Pack-year		12.5 ±7.5	12.9±10.3	0.871
Pirfenidone treatment		23 (95.8)	24 (96.0)	0.976
Initial FVC (L)		2.6 [0.8]	1.6 [1.0]	0.005**
Initial FVC (%)		59.5 [24.9]	38.0 [17.1]	<0.001***
Initial DLCO (%)		39.5 [28.8]	23.0 [14.3]	0.010*
Declined FVC[a] (%)		0.0 [4.9]	13.3 [5.1]	<0.001**
Initial GAP index		4.5 [3.0]	6.0 [2.0]	0.017*
Initial GAP stage		2.0 [2.0]	3.0 [1.0]	0.009**

[a](baseline FVC(L)-FVC(L) at 1 year)/baseline FVC(L)*100

Data presented as mean ± SD, or number (%), or median [IQR]. ″*”, “**” and “***” indicate p<0.05, p<0.01 and p<0.001, respectively. NP: nonprogressive IPF; P: progressive IPF; SD: standard deviation; BMI: body mass index; GAP: gender-age-physiology; FVC: forced vital capacity; DLCO: diffusion capacity of carbon monoxide; IPF: Idiopathic pulmonary fibrosis; IQR: interquartile range.

Table S4. Baseline characteristics of validation cohorts.

	NP (n=90)	P (n=39)	p
Age (years)	68.2±7.4	64.5±7.3	0.011*
Male	68 (75.6)	30 (76.9)	0.867
BMI (kg/m^2)	24.2±3.0	23.4±3.3	0.173
Smoking history			
Never smoking	25 (27.8)	65 (72.2)	0.960
Former smoker	11 (28.2)	28 (71.8)	
Pack-year	11.3±11.8	11.4±13.5	0.974
Pirfenidone treatment	75 (83.3)	32 (82.1)	0.859
Initial FVC (L)	2.8±0.9	2.4±0.9	0.028*
Initial FVC (%)	71.5±16.7	60.1±21.9	0.008**
Initial DLCO (%)	58.3±18.6	43.0±19.7	<0.001***
Declined FVC[a] (%)	3.4 [5.2]	12.9 [6.2]	<0.001***
Initial GAP index	4.0 [1.0]	4.0 [3.0]	0.010*
Initial GAP stage	2.0 [1.0]	2.0 [2.0]	0.008**

[a](baseline FVC(L)-FVC(L) at 1 year)/baseline FVC(L)*100

Data presented as mean±sd or number (%), median [IQR]. "*", "**" and "***" indicate p<0.05, p<0.01 and p<0.001, respectively. NP: nonprogressive IPF; P: progressive IPF; BMI: body mass index, FVC: forced vital capacity, DLCO: diffusion capacity of carbon monoxide, GAP: gender-age-physiology, IPF: Idiopathic pulmonary fibrosis; IQR: interquartile range.

Table S5. CTSB level (ng/ml) according to GAP stage.

	NP (n=90)	P (n=39)	p
**GAP stage**			
**I**	3.5±2.1	10.1±2.1	<0.001***
**II**	3.4±2.3	7.9±1.3	<0.001***
**III**	3.2±2.3	9.0±4.1	<0.001***

″*”, “**” and “***” indicate p<0.05, p<0.01 and p<0.001, respectively. NP: nonprogressive IPF; P: progressive IPF; GAP: gender-age-physiology; IPF: Idiopathic pulmonary fibrosis.

Table S6. ROC curve of CTSB for progressor in each GAP stage.

	AUC	95% CI	p
**GAP stage**			
**I**	0.977	0.95-1.01	<0.001***
**II**	0.927	0.86-0.99	<0.001***
**III**	0.952	0.86-1.04	<0.001***

″*”, “**” and “***” indicate p<0.05, p<0.01 and p<0.001, respectively. AUC: area under the curve, CI: confidence interval, GAP: gender-age-physiology.

Table S7. Summary table of published studies for Hub genes of IPF from 2020 to 2023

Author (year)	Related cohort	Gene expression list	Function
**Leng (2020)**	GSE24206	PPAR, MMP1	signaling pathway, which is involved in cell proliferation and metabolic pathways; drives malignant transformation from IPF to lung cancer
**Zhu (2021)**	GSE10667	VEGFA, CDH5, WNT3A	immune response (GO: antimicrobial humoral response, antimicrobial humoral immune response mediated by antimicrobial peptide, cell killing, organ or tissue specific immune response; GSVA: immunoglobulin complex, antigen processing and presentation of peptide antigen via MHC class Ib, regulation of T-helper 2 cell cytokine production, positive regulation of natural killer cell mediated immunity)
**Li (2020)**	GSE10667, GSE24206	MMP7, TRIM2, ASPN, SULF1, CXCL14, DCLK1, IL13RA2, TP63	ECM-receptor interaction, protein digestion and absorption, and focal adhesion; dilated cardiomyopathy and neuroactive ligand-receptor interaction
**Dai (2022)**	GSE10667, GSE24206	CRTAC1, COL10A1, COMP, IGFL2, NECAB1, SCG5, SLC6A4, SPP1	associated with monocytes, plasma cells, neutrophils, and regulatory (treg) T cells

**Wan (2021)**	GSE10667, GSE24206	COL1A1, COL3A1, CTSK, MMP1, MMP7, POSTN, SPP1	multiple biological processes related to extracellular matrix organization and collagen metabolism by protein digestion and absorption signaling pathways
**Kim (2021)**	GSE10667, GSE24206	CDK1, CDK2	key elements of the p53 signaling pathway and cellular senescence
**Cui (2021)**	GSE10667, GSE24206	ANXA3, STX11, THBS2, MMP1, MMP9, MMP7, MMP10, SPP1, COL1A1, ITGB8, IGF1	protein digestion and PI3k–Akt signaling pathway; pancreatic secretion
**Xia (2020)**	GSE24206	IL6, SOCS3, SERPINE1	inflammation and immune pathways
**Wang (2020)**	GSE24206	lncRNA MALAT1, E2F1, YBX1	cell cycle regulation, methylation, acetyltransferase activity, and the splicing cycle
**Yao (2022)**	GSE24206	COL1A1, COL3A1, MMP1, POSTN1 TIMP3	amoebiasis and relaxin signaling pathways

References

Leng, D., Yi, J., Xiang, M. et al. Identification of common signatures in idiopathic pulmonary fibrosis and lung cancer using gene expression modeling. BMC Cancer 20, 986 (2020). https://doi.org/10.1186/s12885-020-07494-w

Zhu K, Xu A, Xia W, Li P, Han R, Wang E, Zhou S, Wang R. Integrated analysis of the molecular mechanisms in idiopathic pulmonary fibrosis. Int J Med Sci. 2021 Aug 2;18(15):3412-3424. doi: 10.7150/ijms.61309. PMID: 34522168; PMCID: PMC8436110.

Li D, Liu Y, Wang B. Identification of transcriptomic markers for developing idiopathic pulmonary fibrosis: an integrative analysis of gene expression profiles. Int J Clin Exp Pathol. 2020 Jul 1;13(7):1698-1706. PMID: 32782692; PMCID: PMC7414459.

Dai X, Yang Z, Zhang W, Liu S, Zhao Q, Liu T, Chen L, Li L, Wang Y, Shao R. Identification of diagnostic gene biomarkers related to immune infiltration in patients with idiopathic

pulmonary fibrosis based on bioinformatics strategies. Front Med (Lausanne). 2022 Nov 24;9:959010. doi: 10.3389/fmed.2022.959010. PMID: 36507532; PMCID: PMC9729277.

Wan H, Huang X, Cong P, He M, Chen A, Wu T, Dai D, Li W, Gao X, Tian L, Liang H, Xiong L. Identification of Hub Genes and Pathways Associated With Idiopathic Pulmonary Fibrosis via Bioinformatics Analysis. Front Mol Biosci. 2021 Aug 12;8:711239. doi: 10.3389/fmolb.2021.711239. PMID: 34476240; PMCID: PMC8406749.

Kim, S.K., Jung, S.M., Park, KS. et al. Integrative analysis of lung molecular signatures reveals key drivers of idiopathic pulmonary fibrosis. BMC Pulm Med 21, 404 (2021). https://doi.org/10.1186/s12890-021-01749-3

Cui Y, Ji J, Hou J, Tan Y, Han X. Identification of Key Candidate Genes Involved in the Progression of Idiopathic Pulmonary Fibrosis. Molecules. 2021 Feb 20;26(4):1123. doi: 10.3390/molecules26041123. PMID: 33672678; PMCID: PMC7924352.

Xia Y, Lei C, Yang D, Luo H. Identification of key modules and hub genes associated with lung function in idiopathic pulmonary fibrosis. PeerJ. 2020 Sep 8;8:e9848. doi: 10.7717/peerj.9848. PMID: 33194355; PMCID: PMC7485506.

Wang F, Li P, Li FS. Integrated Analysis of a Gene Correlation Network Identifies Critical Regulation of Fibrosis by lncRNAs and TFs in Idiopathic Pulmonary Fibrosis. Biomed Res Int. 2020 Jun 2;2020:6537462. doi: 10.1155/2020/6537462. PMID: 32596346; PMCID: PMC7290873.

Yao Y, Li Z, Gao W. Identification of Hub Genes in Idiopathic Pulmonary Fibrosis and NSCLC Progression:Evidence From Bioinformatics Analysis. Front Genet. 2022 Apr 11;13:855789. doi: 10.3389/fgene.2022.855789. PMID: 35480306; PMCID: PMC9038140.

Table S8. Antibodies and ELISA kit.

		antibodies	ELISA kit
**ADAMTS8**	1:100	No. NBP2-46494; NOVUS Biological	cat. No. MBS763273; MyBioSource, Inc
**GLI2**	1:100	cat. No. ab187386; Abcam; USA	No. MBS9318876; MyBioSource, Inc
**PIK3R2**	1:200	cat. No. ab180967; Abcam; USA	No. MBS109611; MyBioSource, Inc
**CD276**	1:100	cat. No. ab105922; Abcam; USA	No. MBS905704; MyBioSource, Inc
**CTSB**	1:1000	cat. No. ab58802; Abcam; USA	No. ab272205; Abcam; USA
**PRAF2**	1:200	cat. No. ab53113; Abcam; USA	No. MBS280880; MyBioSource, Inc
**COL7A1**	1:100	cat. No. sc-33710; Santa Cruz Biotechnology, Inc	No. MBS763554; MyBioSource, Inc
**IGF2BP3**	1:1000	R&D systems Inc	No. MBS093284; MyBioSource, Inc.
**NUPR1**	1:100	cat. No. 150561-AP; proteintech	None
**TGF beta**			MyBioSource. Cat.No:MBS266143
**IL- 1 beta**			biogems. Cat.No:BGK01584
**IL-6**			MyBioSource. Cat.No:MBS261259
**TNF alpha**			biogems. Cat.No:BGK01375

ADAMTS8: ADAM Metallopeptidase With Thrombospondin Type 1 Motif 8, GLI2: GLI Family Zinc Finger 2, PIK3R2: Phosphoinositide-3-Kinase Regulatory Subunit 2, CD276: Cluster of Differentiation 276, CTSB: Cathepsin B, PRAF2: PRA1 Domain Family Member 2, COL7A1: Collagen Type VII Alpha 1 Chain, IGF2BP3: Insulin Like Growth Factor 2 MRNA Binding Protein 3, NUPR1: Nuclear Protein 1, Transcriptional Regulator, TGF beta: Transforming growth factor beta, IL- 1 beta: Interleukin 1 beta, IL-6: Interleukin 6, TNF alpha: Tumor necrosis factor-alpha.

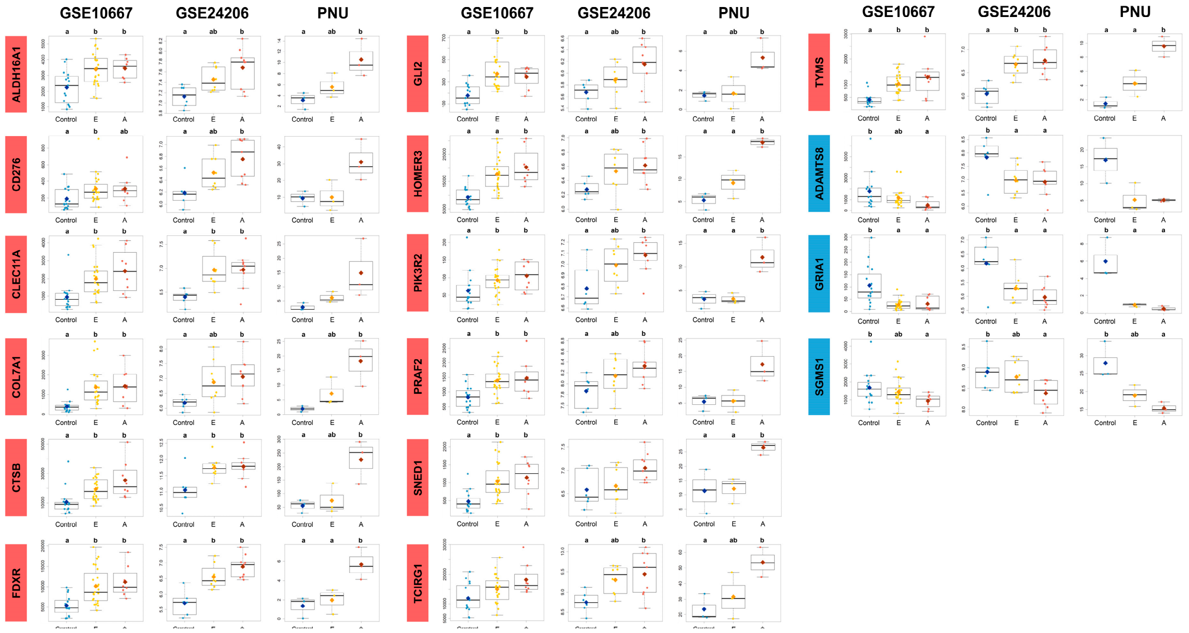

Figure S1. Boxplots showing the expression levels of 16 overlapping DEGs among the three datasets. Red and blue boxes indicate up-regulated and down-regulated genes, respectively. The letters at the top of the boxplots show the result of the Scheffe post hoc test or Bonferroni's multiple comparison test. $^{a-b}$: The same letters in the same column indicate no statistically significant differences. Different letters in the same column indicate significant differences. DEGs: differentially expressed genes; E: early IPF; A: advanced IPF; IPF: Idiopathic pulmonary fibrosis.

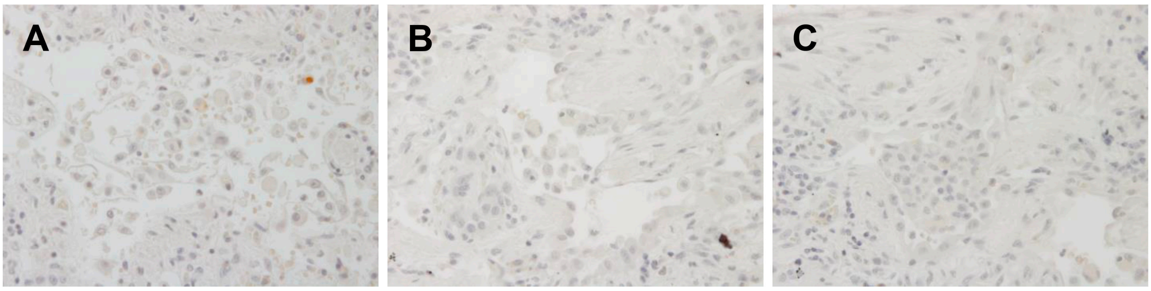

Figure S2. Negative controls for immunohistochemistry.

Representative images show immunostaining for control (A), early IPF (B), and advanced IPF (C).

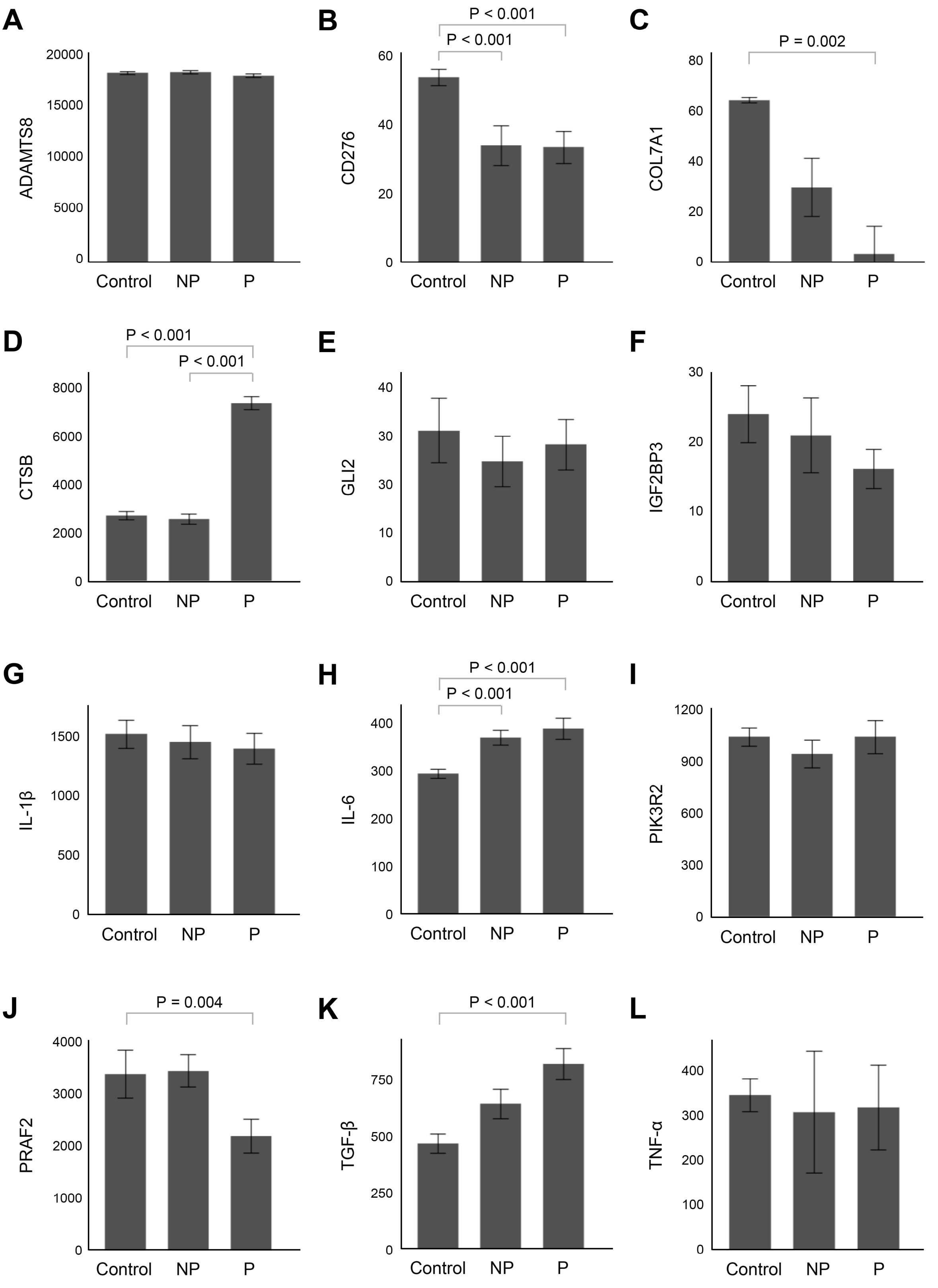

Figure S3. Protein expression of pro-inflammatory cytokines, pro-fibrotic growth factor and differentially expressed genes between the three groups in the test cohorts. Data are

presented as the mean ± 95% CI **(A-J, L)**, or median ± 95% CI **(K)**. There were no significant differences in ADAMTS8, GLI2, IGF2BP3, IL-1β, PIK3R2, TNF-α among the three groups **(A, E-G, I, L)**. IL-6 and TGF-β were significantly higher in the progressive IPF group than in the control group **(H, K)**. NP: nonprogressive IPF; P: progressive IPF; IPF: Idiopathic pulmonary fibrosis.

Supplementary method 1. RNA extraction, library construction, and sequencing

Total RNA from cell samples was extracted using a TRIzol reagent kit (Invitrogen), according to the manufacturer's protocol, and RNA integrity was assessed using a TapStation RNA screentape. After total RNA extraction, RNA libraries were independently prepared using the Illumina TruSeq Stranded Total RNA Library Prep Gold Kit (Illumina, Inc., San Diego, CA, USA). Using random primers, the cleaved RNA fragments were copied into first-strand cDNA using SuperScript II reverse transcriptase (Invitrogen, Carlsbad, CA, USA). Qualified libraries were sequenced on an Illumina NovaSeq platform (Illumina, Inc., San Diego, CA, USA). Total RNA concentration was calculated using the Quant-IT RiboGreen Assay Kit (Invitrogen, Waltham, MA, USA). To determine the values of DV200 (percentage of RNA fragments > 200 bp), samples were run on the TapeStation RNA screentape (Agilent, Wilmington, DE, USA). A total of 100 ng of total RNA was subjected to sequencing library construction using the Agilent SureSelect RNA Direct kit (Agilent, Wilmington, DE, USA) according to the manufacturer's protocol. The total RNA was first fragmented into small pieces using divalent cations at elevated temperatures. The cleaved RNA fragments were copied into first-strand cDNA using random primers. This was followed by second-strand cDNA synthesis. These cDNA fragments then underwent end repair, adding a single "A" base and ligating the adapters. The products were purified and enriched by PCR to create a cDNA library. To capture the human exonic region, the Agilent SureSelect XT Human All Exon v6+UTRs Kit (Agilent, Wilmington, DE, USA) was used according to the standard Agilent SureSelect Target Enrichment protocol. A cDNA library (25 ng) was mixed with hybridization buffers, blocking mixes, RNase block, and 5μL of SureSelect XT Human All Exon v6+UTRs capture library (Agilent, Wilmington, DE, USA). Hybridization to the capture baits was conducted at 65 °C using the heated thermal cycler lid option at 105 °C for 24 h in a PCR machine. The captured

library was washed and subjected to a second round of PCR amplification. The final purified product was then quantified using qPCR according to the qPCR Quantification Protocol Guide (KAPA Library Quantification kits for Illumina Sequencing platforms) and qualified using the TapeStation DNA screentape D1000 (Agilent, Wilmington, DE, USA). The indexed libraries were analyzed using the NovaSeq system (Daejeon, Korea). Macrogen Inc. performed paired-end (2× 100 bp) sequencing.

Supplementary method 2. ELISA and IHC details

A. Immunohistochemistry

Paraffin sections (4 µm) were deparaffinised in xylene and rehydrated in decreasing concentrations of ethanol followed by distilled water. Endogenous peroxidase was quenched with aqueous 3% hydrogen peroxide for 15 min. Antigen retrieval was performed using a pressure cooker filled with 1 mM EDTA buffer (PH 8.0). After incubation with primary antibodies overnight at 4 °C, horseradish peroxidase-conjugated secondary antibody was added for 20 min, followed by 3,3-diaminobenzidine tetrahydrochloride (DAB) for 10 min at room temperature.

B. Western blot

Total protein was denatured by adding Laemmli sample buffer (Bio-Rad, Hercules, CA, USA), 2-mercaptoethanol, and boiling. Fifteen micrograms of total protein were used for immunoblotting. Total protein in the lung tissue was extracted following the manufacturer's instructions (Cat. No. TLP-121.1; TransLab). Protein samples were loaded on 10% sodium dodecyl sulfate–polyacrylamide gel electrophoresis gels, transferred onto polyvinylidene fluoride membranes (Merck Millipore, Germany), blocked with 5% nonfat milk in Tris-buffered saline with 0.1% Tween® 20 Detergent, and incubated with primary antibody at room temperature for 4 h or overnight at 4 °C. The gels were subsequently incubated with horseradish peroxidase-conjugated secondary antibody for one hour. Protein expression was detected by enhanced chemiluminescence (Merck Millipore, Germany). The primary antibody for CTSB is in Table S2 (Cat. No. ab58802; Abcam, Cambridge, USA).
